# Trajectories of depression and anxiety symptom change during psychological therapy

**DOI:** 10.1016/j.jad.2019.02.043

**Published:** 2019-04-15

**Authors:** Rob Saunders, Joshua E.J. Buckman, John Cape, Pasco Fearon, Judy Leibowitz, Stephen Pilling

**Affiliations:** a Research Department of Clinical, Educational and Health Psychology, University College London, Gower Street, London WC1E 7HB, United Kingdom; b iCope – Camden and Islington Psychological Therapies Services, Camden & Islington NHS Foundation Trust, Finsbury Health Centre, Pine Street, London EC1R 0LP, United Kingdom

**Keywords:** Depression, Anxiety, Psychotherapy, IAPT, Latent class growth analysis

## Abstract

**Background:**

Forty-percent of the variance in psychological treatment outcomes is estimated to be explained by symptom change by the third treatment session. However, change may not be uniform across patient groups and symptom domains. This study aimed to identify subgroups of patients with different trajectories of depression and anxiety symptom change during psychological therapy and identify baseline patient characteristics associated with these trajectories.

**Methods:**

4394 patients attending two psychological treatment services completed sessional, self-report depression and anxiety measures. Trajectories of symptom change were investigated using latent class growth analysis. Multinomial logistic regression was used to explore associations between baseline patient characteristics and trajectory classes.

**Results:**

A number of distinct trajectories were identified. Anxiety symptom trajectories could be distinguished by the third treatment session, but for depression symptoms there was a class displaying limited change until session six followed by rapid improvement in symptoms thereafter. Compared to the non-responding trajectories, depression and anxiety trajectories indicating treatment response were associated with lower baseline severity, better social functioning and lower incidence of phobic anxiety, but not with medication prescription status.

**Limitations:**

Data came from two services, so wider generalisability is unknown. Predictors were limited to data routinely collected in the services; unmeasured factors may have improved the prediction of trajectories.

**Conclusions:**

Baseline characteristics and symptom change early in therapy can help identify different trajectories of symptom change. This knowledge could aid clinical decision making and help improve treatment outcomes. By ignoring distinct trajectories, clinicians may incorrectly consider patients as “not-on-track” and unnecessarily change or end therapy that would otherwise benefit patients.

## Highlights

• Four distinct depressive & 5 distinct anxious symptom trajectories were identified.
• Most were distinguishable by session 3 but two depressive trajectories were not.
• Determining “not on track” status at session 3 may lead to missing late-responders.
• Baseline severity, functioning and diagnosis predicted some trajectories.
• Identifying likely trajectories could inform decision-making and optimise care.

## 1 Introduction

Being able to predict the outcome of treatment may be of significant benefit to patients, clinicians and health care services (Lambert et al., 2001a). However, clinicians tend to be poor judges of the probability of patients recovering (Harmon et al., 2007), often overestimating the likelihood of positive outcomes for their own patients (Walfish et al., 2012). Measuring changes in symptom severity during the early stages of treatment has allowed for greater accuracy in predicting treatment outcomes, and has been used to provide clinicians with feedback on the probability of those outcomes to guide their decision making. For example, providing feedback to clinicians about whether their patients are “on track” or “not-on-track” during therapy has led to improved post-treatment outcomes in university based counselling services in the USA (Bybee et al., 2007, Lambert and Shimokawa, 2011). Further, a meta-analysis of controlled studies showed that when feedback based on expected symptom change is provided, more patients recover and the rate of patients experiencing a clinical deterioration during therapy reduces by half among the “not-on-track” patients (Shimokawa et al., 2010).

A consistent pattern of symptom change during psychological treatment has typically shown a rapid improvement between sessions two and four which levels out as the number of treatment sessions increases (Cuijpers et al., 2013, Kopta et al., 1994). Up to 40% of the variance in treatment outcomes has been attributed to symptomatic change by the third therapy session (Lambert, 2013). Meta-analyses of pharmacological interventions have reported similar findings, with relatively large decreases in symptom severity within the first two weeks of treatment being predictive of eventual treatment response (Gorwood et al., 2013, Szegedi et al., 2009).

Overall, outcome feedback systems have demonstrated that identifying standard expected response trajectories and informing clinicians of individual patient's expected treatment response based on these trajectories, can lead to improved patient outcomes. However, the effect sizes from these studies are relatively modest. One potential reason for this is that studies using therapist feedback systems to-date have compared participating patients to an overall averaged response curve across all participants (e.g. Lambert et al., 2001a). It is possible that some patients may follow different trajectories of symptom change, and thus using a singular response curve to compare all patients may reduce the predictive strength of these systems. For example, previous analyses of depression symptom trajectories during a randomised controlled trial of cognitive therapy indicated that although 50% of participants follow a linear, or log-linear function, a small proportion (16%) showed no change before a sudden drop between weeks four and five (Vittengl et al., 2013).

Given that most studies of symptom change to-date have used a cut-point at two weeks (pharmacological) or three sessions (psychological) of treatment, distinguishing between these potentially different groups of patients may not have been possible. If there are distinctly different trajectories of change, identifying these and using them as a basis for comparison should enable clinicians to more accurately predict any new patient's likely prognosis. Further, identifying predictors of the different trajectories of change, at different stages of therapy, ideally at intake, would provide crucial information for guiding treatment decisions.

The identification of patient subgroups for whom trajectories of symptom change differ requires modelling techniques that can identify distinct sub-populations within the sample (Rubel et al., 2015, Spinhoven et al., 2016). Growth mixture modelling (GMM) offers one method of identifying such subgroups (Muthén, 2001, Muthén et al., 2002) and has been used to identify responders (and non-responders) to antidepressant medication (Gueorguieva et al., 2011), as well as classes of change during psychological treatments (e.g. Stulz et al., 2007, Vittengl et al., 2013). More specifically classes have been identified that made sudden symptomatic improvements during cognitive therapy, recruited in a US outpatient psychology service (Tang and DeRubeis, 1999), and early responding trajectories in the first three sessions of psychotherapy delivered across a range of settings (Rubel et al., 2015). However, such techniques have yet to be applied to psychological treatments offered solely within primary care settings, and most previous studies risk being underpowered to reliably detect distinct classes.

The establishment of the Improving Access to Psychological Treatment (IAPT) (Clark, 2011) services in England provides an opportunity to explore trajectories of symptom change in a large naturalistic cohort. The sessional collection of symptom measures is mandatory in these services. IAPT services deliver evidence-based psychological treatments as part of a stepped care model; low-intensity (LI) brief interventions (e.g. self-help) typically delivered for less severe presentations and high-intensity (HI) formal psychological interventions (e.g. cognitive behavioural therapy (CBT)) typically delivered for more severe presentations (Clark, 2018). Approximately 965,000 patients entered IAPT treatment in the 2016–17 financial year (NHS Digital, 2017), and with the numbers of patients treated increasing each year (e.g. Clark, 2018), the ability to predict likely symptom change during IAPT treatments could help optimise service provision. Knowledge regarding recovery and likely post-treatment symptom scores could inform treatment planning, especially when considering that incomplete recovery is a strong predictor of both relapse and returning to the services for further treatment (Ali et al., 2017, Buckman et al., 2018b). Further, following more defined trajectories of symptom change has been found to be associated with better long term outcomes (Vittengl et al., 2016) which could further guide treatment decision making.

The use of feedback to clinicians of the type described in previous studies (e.g. Lambert et al., 2001a), has been tested in a recent multicentre randomised controlled trial conducted in IAPT services (Delgadillo et al., 2018). The study found that when patients were “not-on-track” providing clinicians with feedback helped reduce the risk of poor outcomes compared to patients of clinicians without access to feedback. However, methods to identify distinct trajectories rather than just the mean symptom change may provide greater accuracy in predicting treatment outcomes. Previous studies have reported high correlations between depression and anxiety symptom scores in patients attending treatment services (Ryan et al., 2013) and have suggested that the overall trajectory of depression and anxiety change is similar for adolescents receiving psychological therapy (Queen et al., 2014). However previous research has not explored whether the distinct trajectories of change are similar across symptom measures in adults attending psychological treatment services.

The aims of this study are to model the differing trajectories of change in both depression and anxiety symptoms in patients receiving HI psychological treatments in two psychological treatment services, and to assess whether there are pre-treatment patient characteristics predictive of these trajectories.

## 2 Method

### 2.1 Services

Data were provided by two IAPT services in London. IAPT services provide evidence-based psychological treatments for common mental health disorders such as depression and anxiety disorders. Whilst formal diagnoses are not necessarily made in IAPT services, clinicians are trained to identify what is referred to as the “presenting problem”, which is used as a diagnosis for selecting an appropriate treatment protocol (Clark, 2018). These treatment protocols are recommended by the National Institute for Health and Care Excellence (NICE) and form part of the curriculum for courses training IAPT professionals nationally, meaning all patients with particular conditions should be receiving the same NICE recommended interventions across all IAPT services. Information on treatment protocol adherence is not routinely collected by IAPT services and therefore was not available for the current study, although services are expected to operate their own means of checking adherence through practice and supervision.

### 2.2 Participants

Patients were included in the study if they received HI psychological therapy (e.g. CBT, counselling and interpersonal psychotherapy) between September 2008 and August 2013, provided depressive and anxiety symptom measure scores on at least three occasions, and were above the cut-off for “caseness” on either or both the symptom measure(s) pre-treatment (see measures section below). Patients were excluded if they did not attend at least three sessions of treatment or were below caseness on both symptom severity measures at initial assessment. A minimum of three treatment sessions was required in order to model trajectories of symptom change.

### 2.3 Measures

Patient Health Questionnaire 9-item version (PHQ-9; Kroenke et al., 2001) is a brief measure of depressive symptoms, the cut-off for “caseness” is a score of ≥10. The Generalized Anxiety Disorder Scale 7-item version (GAD-7; Spitzer et al., 2006) is a measure of generalised anxiety disorder symptoms with cut-off for “caseness” of ≥8. Additional information on phobias was collected by the three IAPT phobia scale items (NHS Digital, 2017) and functional impairment was measured using the Work and Social Adjustment Scale (W&SAS: Mundt et al., 2002).

### 2.4 Procedure

Patients completed the self-report symptom measures before every treatment session either by using a secure online portal or by completing them on a pen-and-paper version and handing the completed questionnaires to their clinicians who then entered the scores directly into the electronic patient record system. Analyses were conducted using these sessional data.

### 2.5 Plan of analysis

In distributions with sparse data-points beyond the mean there is a risk that growth curve models can be distorted (Lutz et al., 2005). In this dataset the mean was 10.9 sessions (SD = 4.31) including the baseline assessment, so an upper limit of 13 time points was used including a baseline assessment and 12 therapy sessions.

The first stage of analysis was to identify the average expected response curve for the included sample of patients using latent growth curve analysis (LGC), before identifying distinct sub-groups of patients with differing forms of change using latent class growth analysis (LCGA). LGC methods are used to estimate the mean trajectory of change by pooling all individuals within a sample, incorporating within-person variability (Curran et al., 2010). LGCA is a cluster analytic extension of LGC which categorises individuals with similar patterns of change into subgroups (Lutz et al., 2014). The initial LGC was performed separately for the PHQ-9 and GAD-7; models were built fitting linear and quadratic curves, and by including/excluding residual correlations as a time-varying factor. Model fits were compared using the Comparative Fit Index (CFI) and Tucker-Lewis Index (TFI), alongside the root mean square of error of approximation (RMSEA) and the standardised root mean square residual (SRMR) (Berlin et al., 2014, Geiser, 2013, Schermelleh-Engel et al., 2003).

LCGA models were compared using the following model fit statistics: the Vuong-Lo-Medell-Rubin Likelihood Ratio Test (VLMR-LRT; Lo et al., 2001), the Akaike Information Criterion (AIC), Bayesian Information Criterion (BIC) and entropy values. The VLMR-LRT is a comparison between one model with K-classes, and the K-1 model, with a p-value < 0.05 indicating that the K model fits the data better than the K-1 model. Lower AIC and BIC values for one model compared to another indicate better model fit, whereas higher entropy values indicate higher accuracy in classification for the model.

As there were no prior hypotheses on the number of classes, the LCGA was first conducted with a two-class model, assessing fit statistics and increasing the number of classes until the VLMR-LRT became non-significant or any of the AIC or BIC values increased compared to the previous class solution, as is standard for GMM/LCGA methods (Geiser, 2013, Musliner et al., 2016). Following convention with GMM models, each class had to contain at least 5% of the sample for it to be considered meaningful and numerically stable (Gueorguieva et al., 2011, Spinhoven et al., 2016).

A large number of IAPT patients are expected to be prescribed psychotropic medications (HSCIC, 2014) which may impact symptom change throughout therapy. Therefore, as a secondary analysis the data were split based on medication status at baseline and the best fitting trajectory class solution was modelled on those prescribed medications and those not prescribed medications at baseline.

Finally, multinomial logistic regression models were fitted in order to test associations between the different trajectory classes and patient data routinely collected during the baseline assessment sessions, to consider the possibility of predicting trajectory class membership pre-treatment. Data on each patient's diagnosis/’problem descriptor’ were included as potential predictors. IAPT services collect this information in order to match patients to evidence-based treatments for specific disorders (e.g. trauma-focused CBT for post-traumatic stress disorder) (Clark et al., 2018). Due to distributions of those with each specific problem descriptor, following convention from previous publications using IAPT data from these services (e.g. Buckman et al., 2018a) the problem descriptors were grouped in the following way: firstly, all unipolar depressive disorders were grouped together (32.18% of the included sample); then distinct categories were used for mixed anxiety and depressive disorder (13.59%), generalized anxiety disorder (9.08%), obsessive compulsive disorder (5.58%), and posttraumatic stress disorder (4.01%). All phobic anxiety conditions and panic disorder were grouped together (10.36%), given the low numbers in each category and the routine collection of symptom data related to these disorders with the IAPT phobias scales. Finally, two separate categories were used to denote disorders for which there are not established treatment protocols in IAPT services and for which there are not symptom measures used routinely across all IAPT services (see Clark, 2018). One category was labelled “severe mental illness” (2.46% of the sample) and included psychotic conditions including bi-polar disorder (1.05% of the full sample), schizophrenia (0.46%) and personality disorders (0.96%). These conditions were grouped together due to the likely involvement of additional services and clinicians (e.g. Psychiatrists) in their care. Finally, an “other” category (5.33% of the sample) was created from all remaining conditions for which there are no IAPT treatment protocols and for which there was limited representation to adequately model the specific disorders (e.g. alcohol use disorders [0.30%], eating disorders [0.36%], somatoform disorders [1.21%], bereavement [1.66%] and adjustment disorder [1.80%]).

### 2.6 Missing data, software and packages

LCG and LCGA modelling was performed using Mplus version 7 (Muthén and Muthén, 2012), all regression models were built using Stata version 15 (StataCorp, 2017).

For LCG and LCGA analyses, missing PHQ-9 and GAD-7 data were handled using Full Information Maximum-Likelihood through the Expectation Maximization (EM) algorithm (Dempster et al., 1977) as standard in Mplus. Missing data on potential baseline predictors of PHQ-9 and GAD-7 trajectory classes were imputed using multiple imputation with chained equations using the “ICE” package (Royston and White, 2011).in Stata. All imputation models used the complete data on baseline PHQ-9, GAD-7, W&SAS and the IAPT phobia scales, along with age, gender, ethnicity, and diagnosis. All variables had less than 20% missing data making them eligible for imputation and all imputation models were run 100 times (Royston and White, 2011). The multinomial regressions of baseline variables against the PHQ-9 and GAD-7 trajectory classes (respectively) were run by combining across all 100 imputed datasets.

## 3 Results

### 3.1 Descriptive statistics

Demographic and symptom scores at assessment of the 4394 patients who met inclusion criteria are presented in Table 1. The mean number of treatment sessions attended was 10.56 (SD 4.29). The vast majority (92%) of patients received at least one session of CBT, whereas counselling was received by only 12.06% of patients, and IPT by 1.66% of the sample. All treatments were therefore considered together as High intensity interventions, rather than conducting sub-analyses on specific modalities of intervention.

**Table 1:** Descriptive statistics.

Characteristic	Category	*n*	%
Gender	Female	2783	63.34
	Male	1538	35.00
	Missing	73	1.66
Ethnicity	White	2963	67.43
	Mixed	221	5.03
	Asian	235	5.35
	Black	253	5.76
	Other	172	3.91
	Missing	550	12.52
Prescribed medication	No	1651	37.57
	Yes	1867	42.49
	Missing	876	19.94
Diagnosis	Depression	1414	32.18
	Mixed Anxiety & Depression	597	13.59
	Generalised Anxiety Disorder (GAD)	399	9.08
	Obsessive-compulsive disorder (OCD)	245	5.58
	Post-traumatic stress disorder (PTSD)	176	4.01
	Phobic anxiety & Panic	455	10.36
	Severe mental illness (SMI)	108	2.46
	Other	234	5.33
	Missing	766	17.43
Characteristic	n	Mean (SD)	
Age at referral	4393	38.49 (12.98)	
PHQ-9 score	4394	16.41 (5.65)	
GAD-7 score	4394	14.53 (4.43)	
W&SAS score	4347	21.64 (9.22)	
Social Phobia Score	4354	3.53 (2.64)	
Agoraphobia Score	4352	3.03 (2.81)	
Specific Phobia Score	4350	2.67 (2.81)	
Number of sessions	4394	10.56 (4.29)	

### 3.2 Trajectories of symptom change

**Fig. 1:**
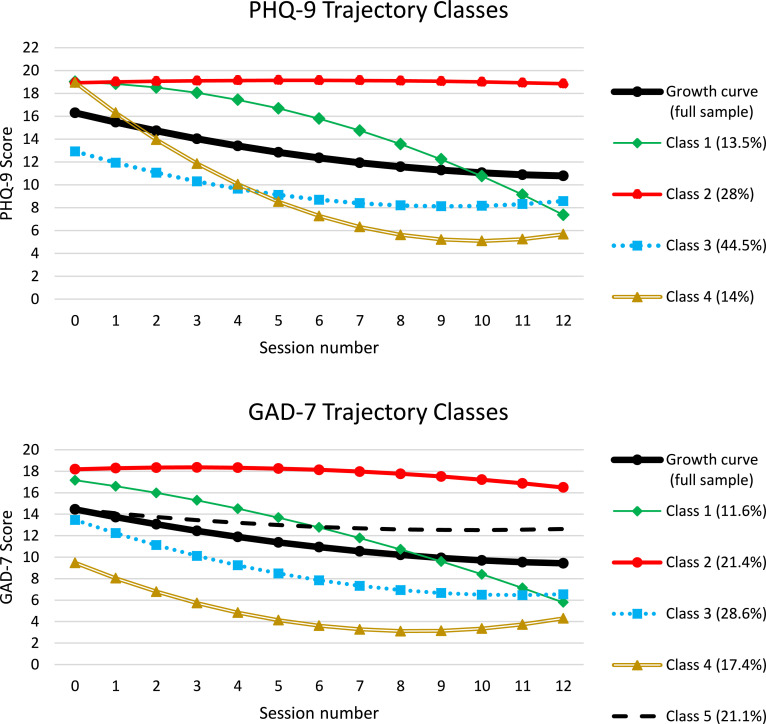
PHQ-9 and GAD-7 trajectory classes.

In the first stage of the analysis, the mean latent growth curve was plotted to estimate the expected response curve on both the PHQ-9 and GAD-7 for the full sample. Model fit statistics are displayed in supplementary table S1. Findings indicated that a linear LGC displayed poor model fit, with CFI and TFI scores below 0.95 for both symptom measures. The introduction of a quadratic factor greatly improved the model fit for both the PHQ-9 (CFI = 0.963, TFI = 0.965) and GAD-7 (CFI = 0.96, TFI = 0.962). The inclusion of correlated residuals resulted in excellent model fit for both measures (PHQ: CFI = 0.988, TFI = 0.987; GAD: CFI = 0.987, TFI = 0.985). The final model trajectories for the PHQ-9 and GAD-7 are presented in Fig. 1 and labelled the “Growth curve (full sample)”, and the growth parameter statistics (mean and 95% confidence intervals) are presented in supplementary table S2. The form of change for both symptom measures indicated a sustained decrease in symptom scores that appeared to slightly level-out as the number of sessions increased.

In the next stage of analysis, LCGA was performed on both measures to identify distinct trajectories beyond the expected response curves. As the LGC analysis identified the model with a quadratic curve and correlated residuals as the best fitting, LCGA was performed using this model specification. Model fit statistics comparing the LGCA of PHQ-9 and GAD-7 are presented in Supplementary material table S3. Model fit of the LCGAs using the parameters stated above led to a 4-class solution for PHQ-9 symptom change and a 5-class solution for GAD-7 symptom change. Growth parameter statistics for the identified trajectories are presented in supplementary table S4.

The identified curves are presented in Fig. 1 alongside the previously identified mean growth curve and the classes can be described as:

PHQ-9:

*Class 1 – Moderate-to-severe pre-treatment, slow initial response, large response later in treatment.
*Class 2 – Moderate-to-severe pre-treatment, limited or no response to treatment.
*Class 3 – Moderate severity pre-treatment, early initial response, levelling out.
*Class 4 – Moderate-to-severe pre-treatment, rapid early improvement, levelling out.

GAD-7:

*Class 1 – Severe pre-treatment, slow initial response, large response later in treatment.
*Class 2 – Severe pre-treatment, limited or no response to treatment.
*Class 3 – Moderate-to-severe pre-treatment, early initial response, levelling out.
*Class 4 – Mild pre-treatment, early initial response, levelling out.
*Class 5 – Moderate-to-severe pre-treatment, limited or no response to treatment.

Differentiation in depressive symptom change trajectories between patients in Class 1 (slow initial response, large response later in treatment) and Class 2 (limited or no response to treatment) appears to occur at around session four with clear differentiation in the trajectories by session six, rather than by session three (as is often reported). Patients in Class 4 (rapid improvement) show almost immediate and sustained response to treatment, representing a group of patients for whom HI treatments can be very effective, and they are distinguishable from patients in the other classes by session 1. The largest class of patients (Class 3; early initial response) has the lowest intercept value (pre-treatment PHQ-9 score), and are distinguishable from those in the other three classes before treatment has begun, and go on to show a reasonable decrease in depression symptoms, which is similar to the mean growth curve of the full sample.

Of the change in anxiety symptoms, GAD-7 Class 1 shares characteristics with PHQ-9 Class 1, in that they have a high pre-treatment score, show limited initial change which gradually increases as treatment sessions continues, and appear distinguishable from the other GAD-7 classes by treatment session 2. Class 2 (Limited response) was similar to PHQ-9 Class 2, both could be defined as non-responders to treatment, with the GAD-7 Class 2 group distinguishable from the other 4 classes at session 2. There was less overlap between GAD-7 classes 1 and 2 compared to PHQ-9 classes 1 and 2, indicating that these GAD-7 classes are distinguishable earlier in therapy than the PHQ-9 classes. The GAD-7 Class 5 appears to be an additional non-responding group of patients, and are differentiated from GAD-7 Class 2 by a lower pre-treatment GAD-7 score. GAD-7 Classes 3 and 4 both appear to be sub-groups of patients who show early initial response to treatment that levels off over time; they are distinguisable from each other pre-treatment, as Class 3 has a considerably higher pre-treatment GAD-7 score than those in Class 4. These two trajectory classes appear to represent lower and higher initial severity versions of PHQ-9 Class 3, although the initial slope of change appears larger in the GAD-7 classes. PHQ-9 Class 4 (rapid response to treatment) does not appear to have a complimentary Class in the GAD-7 analysis, and may indicate that sudden gains are less frequent for anxiety symptoms than depressive symptoms in patients receiving HI IAPT therapies.

The co-occurrences of PHQ-9 and GAD-7 classes within the sample were also explored (see Supplementary material table S5). The largest combinations (34%, *n* = 1484) were for the early initial improvement groups, whereby 20.66% of patients (*n =* 908) were members of PHQ-9 Class 3 and GAD-7 Class 3, and 13.11% (*n* = 576) were members of PHQ-9 Class 3 and GAD-7 Class 4. There was also a large number of individuals who were members of limited response classes on both measures (PHQ-9 Class 2, and GAD-7 Classes 2 or 5), which included nearly 27% (*n* = 1170) of the sample, implying that the lack of response in symptoms on one measure was likely to be reflected by a lack of response on the other.

There were no differences in the form of the trajectories of depressive symptom change between those prescribed (*n* = 1867) and not prescribed (*n* = 1651) medications at baseline, the only difference were that those not prescribed had slightly lower intercept values than those prescribed medications (Supplementary Fig. 1a & b). For anxiety symptom change intercepts were also higher among those prescribed compared to those not prescribed medications. The form of the trajectories for those not prescribed medications was similar to whole sample irrespective of medication status (Supplementary Fig. 1c & d), however for those that were prescribed medications the Class 1 showed a more rapid initial response and lower GAD-7 endpoint, and Class 5 showed a gradual decline in symptoms rather than a lack of response.

### 3.3 Baseline variables associated with trajectories

Associations between baseline patient characteristics and their estimated PHQ-9 and GAD-7 trajectory class (respectively) were analysed in multinomial regression models. Table 2 (PHQ-9) and Table 3 (GAD-7) present odds ratios (OR), 95% confidence intervals (CI) and p-values. Relative to being in PHQ-9 trajectory Class 2 (non-responders): the probability of being in PHQ-9 trajectory Class 1 was higher with higher baseline PHQ-9 scores and lower baseline GAD-7 scores. The probability of being in PHQ-9 trajectory Class 3 relative to class 2 was lower with: lower baseline PHQ-9 scores; lower GAD-7 scores; lower WSAS scores, and lower scores on the IAPT phobia scale items on social phobia and specific phobias; the probability was higher if individuals had a diagnosis of GAD or “Other” diagnoses relative to depression. The probability of being in PHQ-9 trajectory Class 4 compared to Class 2 was higher: with higher PHQ-9 scores; lower WSAS scores; lower scores on the IAPT phobias scale items on social phobia and specific phobias and was higher for those diagnosed with Phobic Anxiety or Panic Disorder, or “Other” diagnoses relative to depression.

**Table 2:** Associations between baseline characteristics and PHQ-9 trajectory classes 1, 3 and 4 relative to class 2 (non-responders).

Baseline predictor	PHQ-9 Class 1 RR(95%CI) & *p*-value	PHQ-9 Class 3 RR(95%CI) & *p*-value	PHQ-9 Class 4 RR(95%CI) & *p*-value
Age	0.88(0.68–1.14), *p* = .332	1.00(0.99–1.00), *p* = .157	1.00(0.99–1.01), *p* = .405
Gender			
Female	0.98(0.77–1.23), *p* = .832	1.04(0.87–1.23), *p* = .677	0.96(0.77–1.19), *p* = .690
Male	1.0	1.0	1.0
Ethnicity			
White	1.0	1.0	1.0
Not white	0.94(0.72–1.24), *p* = .676	0.82(0.66–1.02), *p* = .069	0.90(0.69–1.17), *p* = .420
Primary Diagnosis			
Depression	1.0	1.0	1.0
Mixed A&D	1.21(0.86–1.69), *p *= .275	1.02(0.77–1.33), *p* = .911	0.99(0.70–1.38), *p* = .935
GAD	0.71(0.41–1.24), *p* = .228	1.57(1.23–2.19), *p* = .008	1.35(0.87–2.09), *p* = .176
OCD	1.13(0.67–1.92), *p* = .644	1.00(0.67 = 1.48), *p* = .990	1.04(0.60–1.78), *p* = .895
PTSD	1.03(0.62–1.72), *p* = .622	0.95(0.61–1.47), *p* = .813	1.05(0.63–1.74), *p* = .847
Phobic Anxiety or Panic	1.11(0.73–1.71), *p* = .622	1.34(0.97–1.84), *p* = .073	1.89(0.13–2.81), *p* = .002
Severe MI	0.78(0.37–1.64), *p* = .514	1.29(0.76–2.19), *p* = .337	1.15(0.60–2.20), *p* = .680
Other	0.64(0.33–1.25), *p* = .188	1.65(1.10–2.58), *p* = .015	1.65(1.02–2.66), *p* = .040
Psychotropic Medications			
Prescribed	1.13(0.87–1.46), *p* = .356	0.89(0.73–1.07), *p* = .219	0.80(0.62–1.02), *p* = .066
Not prescribed	1.0	1.0	1.0
PHQ-9 Score	1.04(1.00–1.07), *p* = .028	0.81(0.80–0.83), *p* < .001	1.12(1.08–1.15), *p* < .001
GAD-7 Score	0.97(0.93–1.00), p = .047	0.93(0.91–0.95), p < .001	0.97(0.94–1.00), p = .031
WSAS score	1.00(0.98–1.01), *p* = .706	0.97(0.96–0.98), *p* < .001	0.96(0.95–0.98), *p* < .001
Social Phobia Score	0.96(0.91–1.01), *p* = .128	0.92(0.88–0.96), *p* < .001	0.91(0.86–0.95), *p* < .001
Agoraphobia Score	0.99(0.94–1.05), *p* = .850	0.98(0.94–1.02), *p* = .308	0.97(0.92–1.02), *p* = .237
Specific Phobia Score	0.96(0.92–1.01), *p* = .135	0.95(0.92–0.99), *p* = .007	0.90(0.86–0.94), *p* < .001

**Table 3:** Associations between baseline characteristics and GAD-7 trajectory classes 1, 3, 4, and 5, relative to class 2 (non-responders).

Baseline predictor	GAD-7 Class 1 RR(95%CI) & p-value	GAD-7 Class 3 RR(95%CI) & *p*-value	GAD-7 Class 4 RR(95%CI) & *p*-value	GAD-7 Class 5 RR(95%CI) & *p*-value
Age	0.99(0.98–1.00), *p* = .258	1.0(0.99–1.01), *p* = .992	1.0(0.99–1.01), *p* = .897	1.0(0.99–1.01), *p* = .862
Gender				
Female	0.98(0.76–1.27), *p* = .892	0.83(0.67–1.03), *p* = .093	0.78(0.6–1.01), *p* = .061	0.91(0.73–1.12), *p* = .377
Male	1.0	1.0	1.0	1.0
Ethnicity				
White	1.0	1.0	1.0	1.0
Not white	0.87(0.65–1.18), *p* = .372	0.97(0.76–1.25), *p* = .820	0.76(0.55–1.05), *p* = .094	1.03(0.81–1.32), *p* = .787
Primary Diagnosis				
Depression	1.0	1.0	1.0	1.0
Mixed A&D	1.22(0.84–1.79), *p* = .297	1.05(0.76–1.44), *p* = .761	0.93(0.62–1.4), *p* = .742	1.28(0.93–1.74), *p* = .128
GAD	1.47(0.94–2.30), *p* = .093	1.05(0.71–1.56), *p* = .81	1.18(0.72–1.93), *p* = .51	0.95(0.63–1.43), *p* = .802
OCD	1.20(0.72–2.00), *p* = .474	0.62(0.39–0.98), *p* = .039	0.55(0.3–1), *p* = .052	0.81(0.51–1.28), *p* = .362
PTSD	1.16(0.67–2.00), *p* = .596	0.68(0.41–1.13), *p* = .135	0.87(0.44–1.7), *p* = .682	0.81(0.5–1.31), *p* = .384
Phobic Anxiety or Panic	1.32(0.84–2.08), *p* = .228	1.29(0.89–1.88), *p* = .184	1.26(0.78–2.03), *p* = .349	1.09(0.74–1.61), *p* = .656
Severe MI	1.28(0.63–2.62), *p* = .495	0.8(0.41–1.58), *p* = .524	1.3(0.58–2.91), *p* = .528	1.09(0.58–2.07), *p* = .783
Other	0.95(0.48–1.88), *p* = .890	1.62(0.99–2.65), *p* = .057	1.87(1.02–3.42), *p* = .042	1.62(0.98–2.67), *p* = .06
Psychotropic Medications				
Prescribed	0.84(0.64–1.10), *p* = .196	1.0(0.79–1.26), *p* = .987	0.95(0.71–1.28), *p* = .732	1.01(0.79–1.29), *p* = .926
Not prescribed	1.0	1.0	1.0	1.0
PHQ-9 Score	0.96(0.93–0.99), *p* = .005	0.93(0.91–0.96), *p* < .001	0.92(0.89–0.95), *p* < .001	0.98(0.95–1), *p* = .08
GAD-7 Score	0.94(0.90–0.99), *p* = .012	0.69(0.66–0.71), *p* < .001	0.55(0.52–0.57), *p* < .001	0.71(0.68–0.74), *p* < .001
WSAS score	0.98(0.97–1.00), *p* = .057	0.96(0.95–0.97), *p* < .001	0.95(0.93–0.96), *p* < .001	0.98(0.96–0.99), *p* < .001
Social Phobia Score	0.94(0.89–0.99), *p* = .026	0.93(0.89–0.98), *p* = .004	0.92(0.87–0.98), *p* = .007	0.96(0.91–1.01), *p* = .082
Agoraphobia Score	1.01(0.96–1.07), *p* = .668	0.94(0.9–0.99), *p* = .011	0.89(0.83–0.94), *p* < .001	0.98(0.94–1.03), *p* = .432
Specific Phobia Score	0.93(0.89 = 0.98), *p* = .004	0.94(0.9–0.98), *p* = .004	0.94(0.89–0.99), *p* = .021	0.97(0.93–1.01), *p* = .172

Turning to the results for the GAD-7: relative to being in GAD-7 trajectory class two (non-responders), the probability of being in Class 1 was higher with: lower PHQ-9 scores; lower GAD-7 scores; lower scores on the phobias scale items on social phobia, and specific phobias. The probability of being in GAD-7 trajectory Class 3 compared to Class 2 was higher with lower: PHQ-9 scores; GAD-7 scores; WSAS scores; scores on the three IAPT phobia scale items; it was also lower if diagnosed with OCD relative to depression. The probability of being in GAD-7 trajectory Class 4 was higher with lower PHQ-9 scores, lower GAD-7 scores, lower WSAS scores, lower scores on the three IAPT phobia scales, and if diagnosed with “Other” relative to depression. Finally, the probability of being in GAD-7 trajectory Class 5 relative to Class 2 was higher with lower GAD-7 scores and lower WSAS scores only.

## 4 Discussion

### 4.1 Main findings

This study identified distinct trajectories of change both for symptoms of depression and anxiety in a relatively large cohort of patients receiving high-intensity psychological treatments in two UK psychological treatment services. We found four distinct trajectories of change in depressive symptoms and five distinct trajectories for symptoms of anxiety. Where classes across the symptom domains were similar there was a moderately high degree of overlap such that patients that experienced high degrees of symptomatic change on the PHQ-9 also experienced high degrees of change on the GAD-7. Although like previous studies we found some evidence for a clear distinction between patients being “on-track” or “not-on-track” in psychological therapies by session three, we also found one trajectory class of depression symptoms that showed slow initial response but then rapid later response from session six onwards. In addition, a number of the classes were distinguishable at baseline considering only initial depression or anxiety symptom scores.

### 4.2 Interpretation

The overall treatment responses we observed for both depressive and anxiety symptoms were broadly similar to patterns identified in previous studies (Cuijpers et al., 2013, Kopta et al., 1994). However, when LCGA was used to explore sub-groups of patients based on their symptom patterns over time, we found distinct trajectories of change that differed considerably to the mean response curve. Using these different trajectory classes to inform clinicians of their patients’ progress against the expected response curves might allow for more detailed monitoring and more accurate predictions of treatment outcomes. Further, providing “on-track” or “not-on-track” information based on these trajectories might allow for more informed treatment related decision making and could potentially lead to greater benefits for patients than has been found when basing such feedback on a single growth curve (e.g. Lambert et al., 2001a). This information could be fed-back to clinicians via the electronic health record system giving clinicians and patients an opportunity to consider changing their treatment plan and, with more accurate predictions of treatment outcome, might further inform discussions around extended treatment or relapse prevention interventions (Buckman et al., 2018c).

An interesting finding was that one group of patients showed slow initial response to therapy in their depressive symptoms but later, rapid and sustained improvement, from around session six onwards (PHQ-9 Class 1). There are a number of potential reasons for this: perhaps those in PHQ-9 Class 1 have more complex issues that may take longer to address and affect symptomatic change than those in the early responding class (PHQ-9 Class 3). Alternatively, these patients may be less engaged in the early stages of therapy as the therapeutic relationship which gradually develops as therapy progresses.

If clinicians were able to identify patients who were following this delayed response trajectory, then they might continue with their current treatment plan rather than ending or changing treatment early due to a lack of initial response. This finding goes against the current “conventional wisdom” and findings from previous researchers (Lambert, 2013) which suggests a lack of significant symptomatic improvement by the third therapy session is a signal to the clinician that they should consider changing or stopping treatment. In addition, and of potential utility, baseline characteristics were associated with a higher likelihood of one trajectory over another, for example better responding depressive symptom trajectories were associated with lower depressive symptom severity, lower phobia scores and having GAD or Panic/Phobic anxiety as the presenting problem. Patients in these sub-groups could have their likely prognoses predicted pre-treatment helping inform joint decision making about the potential best care pathways for them. Other classes were distinguishable at session two, slightly earlier than “conventional wisdom” suggests that accurate predictions of prognosis may be made. Further, while previous studies investigating trajectories of change in symptoms during psychological therapies have focussed on single outcome measures (Lambert et al., 2001b) this study identified statistically distinct classes of patients with different trajectories of change in both depression and anxiety symptoms, measured on two different outcome measures, which may be more clinical informative than a single outcome measurement alone.

The trajectories identified in the current analysis have both similarities and differences to previous studies using similar methods in datasets of change in symptoms during acute treatment. A previous analysis of secondary care patients receiving psychological treatment identified five trajectories of symptom change, over the first six sessions of treatment (Stulz et al., 2007), suggesting a similar number of distinct forms of change in alternative datasets. The main differences between the trajectories presented in this paper and that of Stulz and colleagues are that the current trajectories indicated more patients with reductions in symptoms, which may be due to the use of NICE-adherent treatments in IAPT which have proven effectiveness as well as the inclusion of more time points (12 sessions). It is of interest that the current study identified a ‘late responding’ group of patients based on depression symptom change, this group appears to match a group of “one-step” responders identified in a randomised control trial of cognitive therapy for depression (Vittengl et al., 2013, Vittengl et al., 2016). The prevalence of this trajectory was comparable between samples (13% vs 16%) and may indicate a sub-group of patients that exist across a range of settings. Further research to identify this group early on during the therapeutic process could support clinical decision making.

Findings from the current analysis differed from findings by Gueorguiva et al. (2011) who identified only two trajectories from a dataset from a clinical trial of antidepressant treatment. It is possible that the sample used in this analysis is not comparable to the sample used by Gueorguieva et al. (2011) and the more homogenous sampling used in controlled trials may result in less variation in trajectories, or trajectories of change for pharmacological interventions may just differ to those for psychological treatments. Future research would benefit from exploring differences in trajectories between routine treatment datasets and clinical trial datasets, as well as different interventions delivered.

### 4.3 Clinical implications

This paper presents a method of identifying differential responders to psychological treatment for depression and anxiety disorders, using routinely collected patient data. The identification of a sub-population of patients that show slow initial response followed by rapid improvement in depressive symptoms later in therapy, would suggest that continuing treatment beyond the first three sessions even when limited change has been observed could result in a positive outcome from treatment for those in that class. Information about likely trajectory of symptom change, when combined with the use of baseline patient characteristics that can predict likely trajectory class could be used to support clinical decision making and lead to improved treatment outcomes. Being able to identify likely non-responders to therapy at the point of a baseline assessment has several potential implications. Firstly, this might inform joint clinician-patient decision making on whether or not to begin therapy or to consider other treatment options. In addition, if a patient started psychological therapy but their symptoms showed limited change and they were seemingly continuing along the expected trajectory of Class 2, this would be useful information to inform decisions on changing treatment or considering adjunctive options.

### 4.4 Limitations

The dataset used for this analysis comes from two IAPT services in the UK and therefore further analysis with data from additional services would be of value to determine whether these findings are generalizable. Although the baseline characteristics of the patients that make up the present sample are similar to those of other samples of IAPT services from the same time period (e.g. Gyani et al., 2011, Richards and Suckling, 2009), with similar mean PHQ-9 and GAD-7 symptom severity and age, services may differ in treatment decision making practices resulting in differences in patients receiving treatment at high and low intensity across services. All patients in the analysis received High Intensity IAPT interventions which are structured to deliver evidence-based treatment protocols (Clark, 2018). However, information on treatment protocol adherence was not available in the current dataset. Future analyses comparing protocol adherence to trajectories of symptom change could further our ability to use symptom measures to inform clinical decision making. The GAD-7 is limited by its focus just on primarily generalised anxiety symptoms, and other anxiety disorder specific measures may give a more nuanced picture of trajectories of change by different anxiety diagnoses. In addition, data on problem descriptor in IAPT comes from a clinician's judgement of the primary presenting problem and may not have the same prognostic value if clinicians were to use a formalised diagnostic interview. Therefore, results regarding the probability of trajectory class membership differing by problem descriptor must be treated with caution.

The results of the multinomial analyses indicated that compared with the trajectory class of non-responding patients, responding trajectories were associated with lower baseline severity scores and better social functioning. However, the multinomial models identified limited variables (e.g. functional impairment, symptom severity) that were associated with being in the non-responding class and the late responding PHQ-9 class. This may suggest that processes occurring during treatment, such as the nature of therapeutic alliance (Falkenström et al., 2016), may better explain the different likelihood of patients following these trajectories than relying solely on routine patient data collected at baseline. The inclusion of more baseline characteristics that may be associated with outcomes, for example patient treatment preference or information on previous treatments (Cohen and DeRubeis, 2018) that may be available in alternative datasets may also yield better predictions of trajectory membership. Lastly, around 50% of patients providing data had been prescribed psychotropic medication at the time of assessment, but no information was available on the details of their prescription nor on their adherence to this. Although being prescribed medication was not significantly associated with any trajectory, including data on adherence to medication could be informative and therefore of value to explore in future research. It is noteworthy that trajectories of change for depressive symptoms were very similar whether or not patients were prescribed medication at baseline, and two classes of anxiety symptom change showed greater degrees of response among those prescribed medications at baseline.

## 5 Conclusions

This study adds to the literature identifying distinct forms of change in symptoms during treatment for common mental disorders by using growth mixture modelling techniques in a large sample of patients receiving psychological therapy in routine treatment services. The sample size benefits allow for reliable detection of distinct classes and support a number of previous findings in other settings. We identified a number of distinct trajectories of change in symptom scores, indicating different prognoses following treatment for distinct subgroups of those receiving psychological therapies. Most of these trajectories could be differentiated by the third session of treatment, consistent with previous research. However, one depression symptom trajectory showed slow initial response followed by a rapid later improvement in symptoms. Further, a number of patient characteristics measured pre-treatment were associated with greater likelihood of certain trajectories, giving the opportunity to consider likely prognosis before treatment begins. The consideration of these trajectories could be used to inform clinicians about the likely level of symptoms at the end of treatment, helping to differentiate when changes to a treatment regimen may be required.

## 6 Contributors

RS and SP conceived the original idea for this review with support from JB, PF and JC. RS prepared the data for analysis and conducted the latent growth curve modelling; RS & JB performed the multinomial regression analyses. RS and JB prepared the manuscript with repeated revisions commented on and amended by JC, PF, JL and SP.

## 7 Role of the funding source

This work was supported by grants from the National Institute for Health Research University College London Hospitals Biomedical Research Centre, the Wellcome Trust (Grant Code 201292/Z/16/Z) and NoClor (Camden & Islington NHS Foundation Trust). None of these funders had any role in the study design, collection, analysis or interpretation of the data, writing the manuscript, or the decision to submit the paper for publication

## Appendix Supplementary materials

Image, application 1
