# Supplementary material for: Trajectories of depression and anxiety symptom change during psychological therapy
Source: J Affect Disord. 2019 Apr 15;249:327–35. doi: 10.1016/j.jad.2019.02.043 (PMC6428692; doi:10.1016/j.jad.2019.02.043)
Supplement: Supplementary file 1 [file mmc1.docx]

**Supplementary online content:**

**Supplementary Table S1.** Latent growth curve model comparison.

**Supplementary Table S2.** Growth parameter statistics: latent growth curve models.

**Supplementary Table S3.**LGCA Model fit statistics.

**Supplementary Table S4.**Growth parameter statistics: final LGCA models.

**Supplementary Table S5.** Co-occurrence of PHQ-9 and GAD-7 Trajectory classes.

**Supplementary Figure S1.** PHQ-9 and GAD-7 trajectory classes: Comparison between patients prescribed and not prescribed medication at baseline.

**Supplementary Table S1. Latent growth curve model comparison.**

|  | **PHQ-9** | | | | **GAD-7** | | | |
| --- | --- | --- | --- | --- | --- | --- | --- | --- |
|  | CFI | TFI | RMSEA | SRMR | CFI | TFI | RMSEA | SRMR |
| **Linear** | 0.915 | 0.923 | 0.07 | 0.1 | 0.9 | 0.909 | 0.073 | 0.118 |
| **Quadratic** | 0.963 | 0.965 | 0.047 | 0.04 | 0.96 | 0.962 | 0.047 | 0.045 |
| **Quadratic(correlated residuals)** | 0.988 | 0.987 | 0.029 | 0.028 | 0.987 | 0.985 | 0.029 | 0.034 |

**Supplementary Table S2****. Growth parameter statistics: latent growth curve models.**

| **PHQ-9** | | | | | | |
| --- | --- | --- | --- | --- | --- | --- |
|  | **Intercept** | | **Linear** | | **Quadratic** | |
| **Overall mean (full sample)** | Mean | 95% Cis | Mean | 95% Cis | Mean | 95% Cis |
|  | 16.309 | (16.14 to 16.48) | -0.855 | (-0.9 to -0.81) | 0.033 | (0.03 to 0.04) |
| **GAD-7** | | | | | | |
|  | **Intercept** | | **Linear** | | **Quadratic** | |
| **Overall mean (full sample)** | Mean | 95% Cis | Mean | 95% Cis | Mean | 95% Cis |
|  | 14.460 | (14.32 to 14.6) | -0.755 | (-0.8 to -0.71) | 0.028 | (0.02 to 0.03) |

**Supplementary Table S3LGCA Model fit statistics**.

| **PHQ-9** | | | | | | |
| --- | --- | --- | --- | --- | --- | --- |
| **k model** | **AIC** | **BIC** | **Adj-BIC** | **VLMR-LRT (p=)** | **Entropy** | **% individuals in per class** |
| k = 2 | 198731 | 198942 | 198837 | <0.001 | 0.556 | 39/61 |
| k = 3 | 198254 | 198490 | 198373 | 0.002 | 0.557 | 33/45/22 |
| **k = 4** | **197868** | **198130** | **197999** | **<0.001** | **0.598** | **45/13/14/28** |
| k = 5 | 197759 | 198046 | 197903 | 0.011 | 0.629 | 3/14/43/13/28 |
| k = 6 | 197701 | 198014 | 197858 | 0.438 | 0.585 | 22/28/13/13/20/3 |
| **GAD-7** | | | | | | |
| **k model** | **AIC** | **BIC** | **Adj-BIC** | **VLMR-LRT (p=)** | **Entropy** | **% individuals in per class** |
| k = 2 | 195067 | 195271 | 195169 | <0.001 | 0.847 | 53/47 |
| k = 3 | 191764 | 191994 | 191879 | <0.001 | 0.824 | 41/27/32 |
| k = 4 | 190762 | 191018 | 190891 | <0.001 | 0.772 | 21/30/30/19 |
| **k = 5** | **190440** | **190721** | **190581** | **0.039** | **0.739** | **21/29/17/21/12** |
| k = 6 | 190064 | 190371 | 190218 | 0.375 | 0.715 | 20/12/13/20/18/17 |

**Supplementary Table S4.Growth parameter statistics: final LGCA models**.

| **PHQ-9 LCGA classes** | | | | | | |
| --- | --- | --- | --- | --- | --- | --- |
|  | **Intercept** | | **Linear** | | **Quadratic** | |
|  | Mean | 95% Cis | Mean | 95% Cis | Mean | 95% Cis |
| Class 1 | 12.929 | (12.51 to 13.35) | -1.046 | (-1.16 to -0.93) | 0.057 | (0.05 to 0.06) |
| Class 2 | 19.025 | (18.34 to 19.71) | -0.106 | (-0.37 to 0.15) | -0.072 | (-0.09 to -0.05) |
| Class 3 | 18.968 | (18.26 to 19.68) | -2.787 | (-3.05 to -2.52) | 0.140 | (0.12 to 0.16) |
| Class 4 | 18.944 | (18.5 to 19.39) | 0.076 | (-0.02 to 0.17) | -0.007 | (-0.01 to 0) |
| **GAD-7 LCGA classes** | | | | | | |
|  | **Intercept** | | **Linear** | | **Quadratic** | |
|  | Mean | 95% Cis | Mean | 95% Cis | Mean | 95% Cis |
| Class 1 | 18.190 | (17.96 to 18.42) | 0.123 | (0.05 to 0.2) | -0.022 | (-0.03 to -0.02) |
| Class 2 | 13.472 | (12.91 to 14.04) | -1.297 | (-1.45 to -1.14) | 0.060 | (0.05 to 0.07) |
| Class 3 | 9.479 | (8.95 to 10.01) | -1.523 | (-1.66 to -1.39) | 0.091 | (0.08 to 0.1) |
| Class 4 | 14.457 | (13.55 to 15.36) | -0.393 | (-0.52 to -0.26) | 0.020 | (0.01 to 0.03) |
| Class 5 | 17.164 | (16.66 to 17.66) | -0.516 | (-0.81 to -0.22) | -0.036 | (-0.06 to -0.02) |

**Supplementary Table S5. Co-occurrence of PHQ-9 and GAD-7 Trajectory classes.**

| **PHQ-9 Class** | **GAD-7 Class** | | | | |
| --- | --- | --- | --- | --- | --- |
|  | **1 - Slow initial** | **2 - Limited response (severe)** | **3 - Early initial (severe)** | **4 - Early initial (low)** | **5 - Limited response (low)** |
| **1 - Slow initial** | 3.48% | 2.00% | 1.87% | 0.27% | 1.82% |
| **2 - Limited response** | 0.68% | 17.57% | 1.98% | 0.30% | 9.06% |
| **3 - Early initial** | 3.14% | 2.41% | 20.66% | 13.11% | 9.65% |
| **4 - Rapid improvement** | 2.00% | 0.36% | 5.26% | 3.48% | 0.89% |

**Supplementary Figure S1.** PHQ-9 and GAD-7 trajectory classes: Comparison between patients prescribed and not prescribed medication at baseline.

Figures S1a & S1b: Trajectory classes for patients not prescribed medication at baseline

Figures S1c & S1d: Trajectory classes for patients who were prescribed medication at baseline
